# Prenatal Exposure to Gabapentin Alters the Development of Ventral Midbrain Dopaminergic Neurons

**DOI:** 10.3389/fphar.2022.923113

**Published:** 2022-07-22

**Authors:** Walaa F. Alsanie, Sherin Abdelrahman, Majid Alhomrani, Ahmed Gaber, Hamza Habeeballah, Heba A. Alkhatabi, Raed I. Felimban, Charlotte A. E. Hauser, Hossam H. Tayeb, Abdulhakeem S. Alamri, Bassem M. Raafat, Sirajudheen Anwar, Khaled A. Alswat, Yusuf S. Althobaiti, Yousif A. Asiri

**Affiliations:** ^1^ Department of Clinical Laboratories Sciences, The Faculty of Applied Medical Sciences, Taif University, Taif, Saudi Arabia; ^2^ Centre of Biomedical Sciences Research (CBSR), Deanship of Scientific Research, Taif University, Taif, Saudi Arabia; ^3^ Laboratory for Nanomedicine, Division of Biological and Environmental Science and Engineering (BESE), King Abdullah University of Science and Technology (KAUST), Jeddah, Saudi Arabia; ^4^ Computational Bioscience Research Center (CBRC), King Abdullah University of Science and Technology, (KAUST), Jeddah, Saudi Arabia; ^5^ Department of Biology, College of Science, Taif University, Taif, Saudi Arabia; ^6^ Department of Medical Laboratory Technology, Faculty of Applied Medical Sciences in Rabigh, King Abdulaziz University, Jeddah, Saudi Arabia; ^7^ Department of Medical Laboratory Sciences, Faculty of Applied Medical Sciences, King Abdulaziz University, Jeddah, Saudi Arabia; ^8^ Center of Excellence in Genomic Medicine Research (CEGMR), King Abdulaziz University, Jeddah, Saudi Arabia; ^9^ King Fahd Medical Research Centre, Hematology Research Unit, King Abdulaziz University, Jeddah, Saudi Arabia; ^10^ Center of Innovation in Personalized Medicine (CIPM), 3D Bioprinting Unit, King Abdulaziz University (KAUST), Jeddah, Saudi Arabia; ^11^ Center of Innovation in Personalized Medicine (CIPM), Nanomedicine Unit, King Abdulaziz University, Jeddah, Saudi Arabia; ^12^ Department of Radiological Sciences, College of Applied Medical Sciences, Taif University, Taif, Saudi Arabia; ^13^ Department of Pharmacology and Toxicology, College of Pharmacy, University of Hail, Hail, Saudi Arabia; ^14^ Department of Internal Medicine, School of Medicine, Taif University, Taif, Saudi Arabia; ^15^ Department of Pharmacology and Toxicology, College of Pharmacy, Taif University, Taif, Saudi Arabia; ^16^ Addiction and Neuroscience Research Unit, Taif University, Taif, Saudi Arabia; ^17^ Department of Clinical Pharmacy, College of Pharmacy, Taif University, Taif, Saudi Arabia

**Keywords:** gabapentin, neurons, morphogenesis, pregnancy, development

## Abstract

**Background:** Gabapentin is widely prescribed as an off-label drug for the treatment of various diseases, including drug and alcohol addiction. Approximately 83–95% of the usage of gabapentin is off-label, accounting for more than 90% of its sales in the market, which indicates an alarming situation of drug abuse. Such misuse of gabapentin has serious negative consequences. The safety of the use of gabapentin in pregnant women has always been a serious issue, as gabapentin can cross placental barriers. The impact of gabapentin on brain development in the fetus is not sufficiently investigated, which poses difficulties in clinical decisions regarding prescriptions.

**Methods:** The consequences effect of prenatal gabapentin exposure on the development of ventral midbrain dopaminergic neurons were investigated using three-dimensional neuronal cell cultures. Time-mated Swiss mice were used to isolate embryos. The ventral third of the midbrain was removed and used to enrich the dopaminergic population in 3D cell cultures that were subsequently exposed to gabapentin. The effects of gabapentin on the viability, ATP release, morphogenesis and genes expression of ventral midbrain dopaminergic neurons were investigated.

**Results:** Gabapentin treatment at the therapeutic level interfered with the neurogenesis and morphogenesis of vmDA neurons in the fetal brain by causing changes in morphology and alterations in the expression of key developmental genes, such as *Nurr1, Chl1, En1, Bdnf, Drd2,* and *Pitx3.* The TH + total neurite length and dominant neurite length were significantly altered. We also found that gabapentin could halt the metabolic state of these neuronal cells by blocking the generation of ATP.

**Conclusion:** Our findings clearly indicate that gabapentin hampers the morphogenesis and development of dopaminergic neurons. This implies that the use of gabapentin could lead to serious complications in child-bearing women. Therefore, caution must be exercised in clinical decisions regarding the prescription of gabapentin in pregnant women.

## Introduction

Gabapentin is most commonly prescribed to treat neuropathic pain. Gabapentin is a structural analog of the inhibitory neurotransmitter gamma-aminobutyric acid (GABA). Gabapentin does not directly act upon GABA receptors; however, it has a tendency to increase GABA and decrease the levels of glutamate neurotransmitters (Smith et al., 2016). The mechanism through which gabapentin acts is not clear; however, it has been reported that gabapentin may decrease the release of pain-related peptides and reduce opioid-induced hyperalgesia (Compton et al., 2010). Gabapentin can also bind to protein subunits of the voltage-dependent Ca^++^ channel complex, suggesting that it can modulate neuron signaling (Suman-Chauhan et al., 1993; Thurlow et al., 1993; Gee et al., 1996). Gabapentin was first approved for epilepsy in 1993. Later, in 2004, it was also authorized to treat postherpetic neuralgia (Mack, 2003). In 2006, UK regulatory agencies approved it as a first-line treatment for all types of neuropathic pain (Smith et al., 2016). Gabapentin is widely prescribed as an off-label drug to treat several diseases, including drug and alcohol addiction, insomnia, bipolar disorder, borderline neuropathic pain conditions, pruritic disorders, anxiety, menopausal conditions, migraines, and vertigo. Approximately 83–95% of the usage of gabapentin is off-label, which accounts for more than 90% of its sales in the market (Hamer et al., 2002; Radley et al., 2006). Pfizer was penalized a fine of $420 million for illegal marketing of gabapentin with off-label uses (Newman, 2010).

There is a growing concern about the misuse of gabapentin as it can produce a euphoria and anxiolytic effects similar to recreational drugs. The misuse of gabapentin is known to have serious consequences. There are growing concerns regarding the use of gabapentin during pregnancy because of the lack of clarity regarding the effects of gabapentin on brain development. The ability of gabapentin to cross the placental barrier has made the safety of child-bearing women a serious issue. It is still unclear whether the therapeutic dose of gabapentin impairs brain development in the fetus (Patorno et al., 2020). Considering the lack of convincing evidence concerning the impact of gabapentin on fetal brain development during pregnancy, we used embryonic ventral midbrain (VM) neurons of mice to investigate the effects of gabapentin on brain development by examining protein expression and morphological characteristics of ventral midbrain dopaminergic (vmDA) neurons, which play vital roles in controlling cognition and motor activities. In addition, vmDA neurons are involved in learning, motivation, reward association and regulation of movement. In the present study, we show for the first time the effects of gabapentin exposure on the development of vmDA neurons isolated from mouse embryos.

## Materials and Methods

### Separation of Primary Dopaminergic Neurons From the Ventral Midbrain of a Mouse Embryo

**FIGURE 1:**
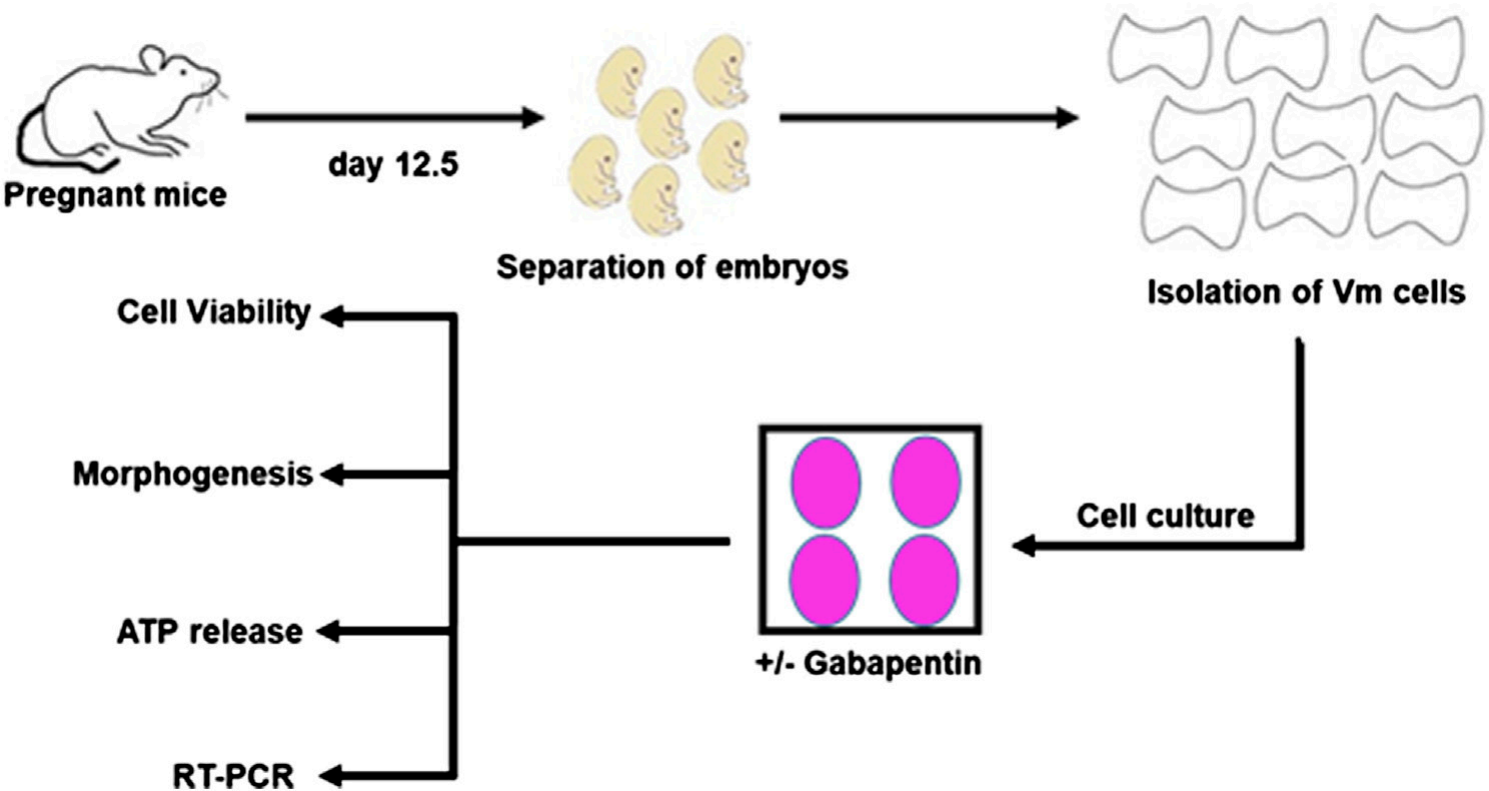
The experimental design of the present study.

The experiments were approved by the King Abdulaziz University (12-CEGMR-Bioethic-2020) and Taif University (211BEC023) ethics committees, and they followed international norms for the use of animals in research. Figure 1 explains the experimental technique. Time-mated Swiss mice were used to isolate embryos. Animals were time-mated overnight, and the next morning’s sight of a vaginal plug was termed as embryonic day (E) 0.5. In ice-cold L-15 Medium (Thermo Fisher, USA), the VM of E12.5 mouse embryos was dissected. E12.5 mouse embryos have been used as it is the optimum time point for analyzing the effects of external signaling cues on developing vmDA neurons. To isolate the midbrain and cortical tissues, the telencephalon–mesencephalon boundary as well as the isthmic organizer were separated. The dopaminergic population in the culture was enriched by dissecting the ventral third of the midbrain tissue. The separated VMs were incubated for 15 min at 37 °C in a mixture of 0.05 percent trypsin (Thermo Fisher Scientific, USA) and 0.1 percent DNase (STEMCELL Technologies, USA) diluted in Ca/Mg-free Hank’s Balanced Salt Solution (HBSS) (Thermo Fisher Scientific, USA). The tissues were washed three times in HBSS before being resuspended in N2 media, which was made up of a 1:1 mixture of F12 medium and Minimum Essential Medium enhanced with 1 mM glutamine, 1 mg/ml BSA, 15 mM HEPES, 6 mg/ml glucose, 1% penicillin/streptomycin, and 1% N2 supplement. Primary neurons were cultured for three days before investigation

### Neuronal Cultures

We used three-dimensional (3D) neural cell cultures to evaluate cell survival, ATP release, and morphogenesis, as well as perform real-time PCR. Freshly isolated primary embryonic mouse E12.5 vmDA neurons were sown at a density of 6 × 104 cells/well to create the 3D culture. The short peptide IIZK was then used as described in a previous publication (Alsanie et al., 2020). IIZK was prepared in 1 × Dulbecco’s phosphate-buffered saline (DPBS). The weighted IIZK peptide was resuspended in half of the needed final volume of nuclease-free sterile water. Due to the possibility of quick gelation when using DPBS, it was only used inside the cell culture well. To aid the gelation process, an appropriate volume of previously resuspended peptide in water was introduced to the culture well, followed by an equivalent volume of 2 × DPBS. To prevent cells from coming into contact with the plastic surface, a peptide base was initially added to each well. In order to achieve complete gelation, the plates were incubated for 5 min. A 3D construct was then developed on top of the peptide basal layer. The addition of peptides was followed by that of an equivalent volume of 2× DPBS containing the desired number of cells, which was then mixed briefly with the peptide. Before adding cell culture media, the plates were re-incubated for 2–3 min. The culture plates were then properly filled with N2 media. Next, the cells were incubated for 72 h. Gabapentin solution was made according to the manufacturer’s guidelines (Sigma-Aldrich, USA). Gabapentin with a final concentration of 10 µM was administered to the gabapentin treatment group immediately after seeding the cells, whereas the control groups received an identical volume of sterile 1× PBS. The 10 µM gabapentin dose was used in the current study to resemble the serum concentration levels in the therapeutic dose.

### Viability and ATP Release of vmDA Neurons

After three days of culture, the change in ATP release and viability of E12.5 vmDA neuron in response to gabapentin treatment were measured by alamarBlueTM Cell Viability Reagent (Thermo Fisher, USA) according to the manufacturer’s guidelines. The fluorescence was measured using a PHERAstar FS (BMG LabTech, Germany). The CellTiter-Glo^®^ 3D Cell Viability Assay (Promega, G9681) was used to determine the status of the metabolic activity and ATP release of the cells. The luminescent signal was read by PHERAstar FS plate reader (BMG LabTech, Germany).

### Immunocytochemistry

After three days of culturing, the mouse embryonic vmDA neurons were fixed with 4% paraformaldehyde (Santa Cruz Biotechnology, sc-281692) and kept at 4°C in 1× PBS before staining. The immunocytochemistry experiment were carried out using primary antibodies against mouse neuron-specific class III beta-tubulin (TUJ1) (Promega, G7121) and tyrosine hydroxylase (TH) (Abcam, ab112). The primary antibodies TUJ1 (1:1500) and TH (1:500) diluted in blocking buffer and incubated overnight at room temperature with fixed cultures. After removing the primary antibodies, the cells were treated in blocking buffer for 1 h at room temperature. The secondary antibodies anti-mouse Alexa 488 and goat anti-rabbit IgG H&L (Alexa Fluor^®^ 555) (Abcam, ab150078) were then added and incubated for 2 h at room temperature, diluted in blocking buffer (1:200). The cells were then rinsed and kept in 1× PBS after being treated for 5 min in 4′,6-diamidino-2-phenylindole, dihydrochloride (DAPI) (Thermo Fisher Scientific, D1306). A DMi8 inverted fluorescent microscope was used for imaging (Leica, Germany).

### Morphogenesis

In stained cultures, the impacts of gabapentin on the development of VM neurons were investigated. The LAS X software (Leica, Germany) was used to assess the number of neurites, total length of neurites, length of dominant neurites, and number of branches, as detailed previously (Alsanie et al., 2017). To eliminate bias, overlapping neurites and those shorter than 20 µm were omitted. The results from the gabapentin-treated cultures were compared to the control group’s results. Following that, the data were reported as a percentage change from the control values, which were assumed to be 100%.

### Real-Time PCR

The RNeasy Plus universal Mini Kit (Qiagen, Cat no. 73404) was used to isolate RNA from the VM neurons in gabapentin-treated and control cultures after three days of culturing. TissueLyser II (Qiagen) was employed as suggested in the RNeasy kit protocol to guarantee optimal cell homogenization. A negative control was RNA obtained from mouse tissues other than brain tissues. Table 1 shows the primer sequences for the designated genes. The StepOneTM Real-Time PCR System and DataAssist software (Thermo Fisher) were used to generate raw threshold cycle (CT) data for all selected genes (Table 1). The experiments were performed triplicates. The expression of each gene was measured by normalizing it to a housekeeping gene (*Gapdh*). The ∆∆CT method was used to calculate RNA expression. The expression levels of each gene were compared across all five groups’ samples, and *p* values were calculated to identify genes that were significantly expressed.

**TABLE 1:** Gene-specific primer pair sequences used in the RT–PCR.

Genes Name	Primer sequence (5′–3′)
Gapdh	Forward: TGA AGG TCG GAG TCA ACG GA
	Reverse: CCA ATT GAT GAC AAG CTT CCC G
Th	Forward: TGA AGG AAC GGA CTG GCT TC
	Reverse: GAG TGC ATA GGT GAG GAG GC
Nurr1	Forward: GAC CAG GAC CTG CTT TTT GA
	Reverse: ACC CCA TTG CAA AAG ATG AG
Lmx1a	Forward: GAG ACC ACC TGC TTC TAC CG
	Reverse: GCA CGC ATG ACA AAC TCA TT
En1	Forward: TCA CAG CAA CCC CTA GTG TG
	Reverse: CGC TTG TCT TCC TTC TCG TT
Pitx3	Forward: CAT GGA GTT TGG GCT GCT TG
	Reverse: CCT TCT CCG AGT CAC TGT GC
Chl1	Forward: TGG AAT TGC CAT TAT GTG GA
	Reverse: CAC CTG CAC GTA TGA CTG CT
Dat	Forward: TTG CAG CTG GCA CAT CTA TC
	Reverse: ATG CTG ACC ACG ACC ACA TA
Drd2	Forward: CTC AAC AAC ACA GAC CAG AAT
	Reverse: GAA CGA GAC GAT GGA GGA
Bdnf	Forward: ACT ATG GTT ATT TCA TAC TTC GGT T
	Reverse: CCA TTC ACG CTC TCC AGA

### Statistical Analysis

The data were analyzed using the Student’s t test performed using GraphPad Prism v 8.1.2. All quantitative data were expressed as the mean ± SEM with the significance level set at *p* < 0.05.

## Results

### Gabapentin Disrupts the Metabolic Activity of vmDA Neurons

**FIGURE 2:**
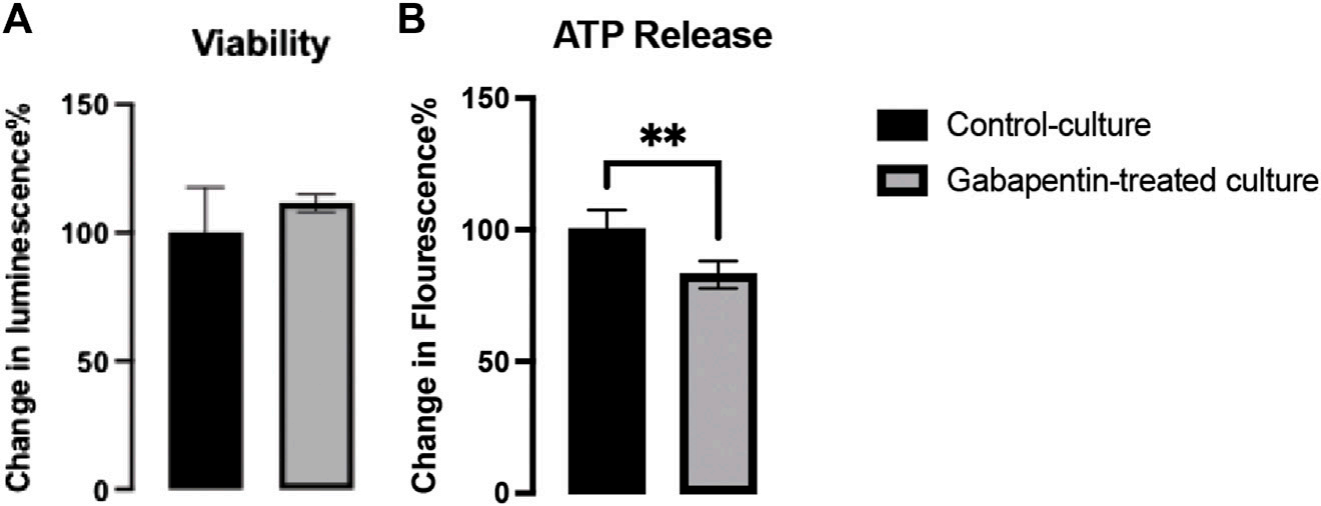
Viability **(A)** and ATP release **(B)** in gabapentin-treated and control cultures.

Neurotoxicity caused by gabapentin has been reported to occur in cases of renal failure, despite dose adjustment (Wahba and Waln, 2013). Our results showed that gabapentin did not have any impact on the viability of neurons (Figure 2A), thus confirming that it is nontoxic at therapeutic doses. However, we found a significant decrease in ATP generation in gabapentin-treated cultures. It can be thus inferred that gabapentin interferes with the mitochondrial electron transport system, causing metabolic dysfunction (Figure 2B).

### Gabapentin Affects the Morphogenesis of vmDA Neurons

**FIGURE 3:**
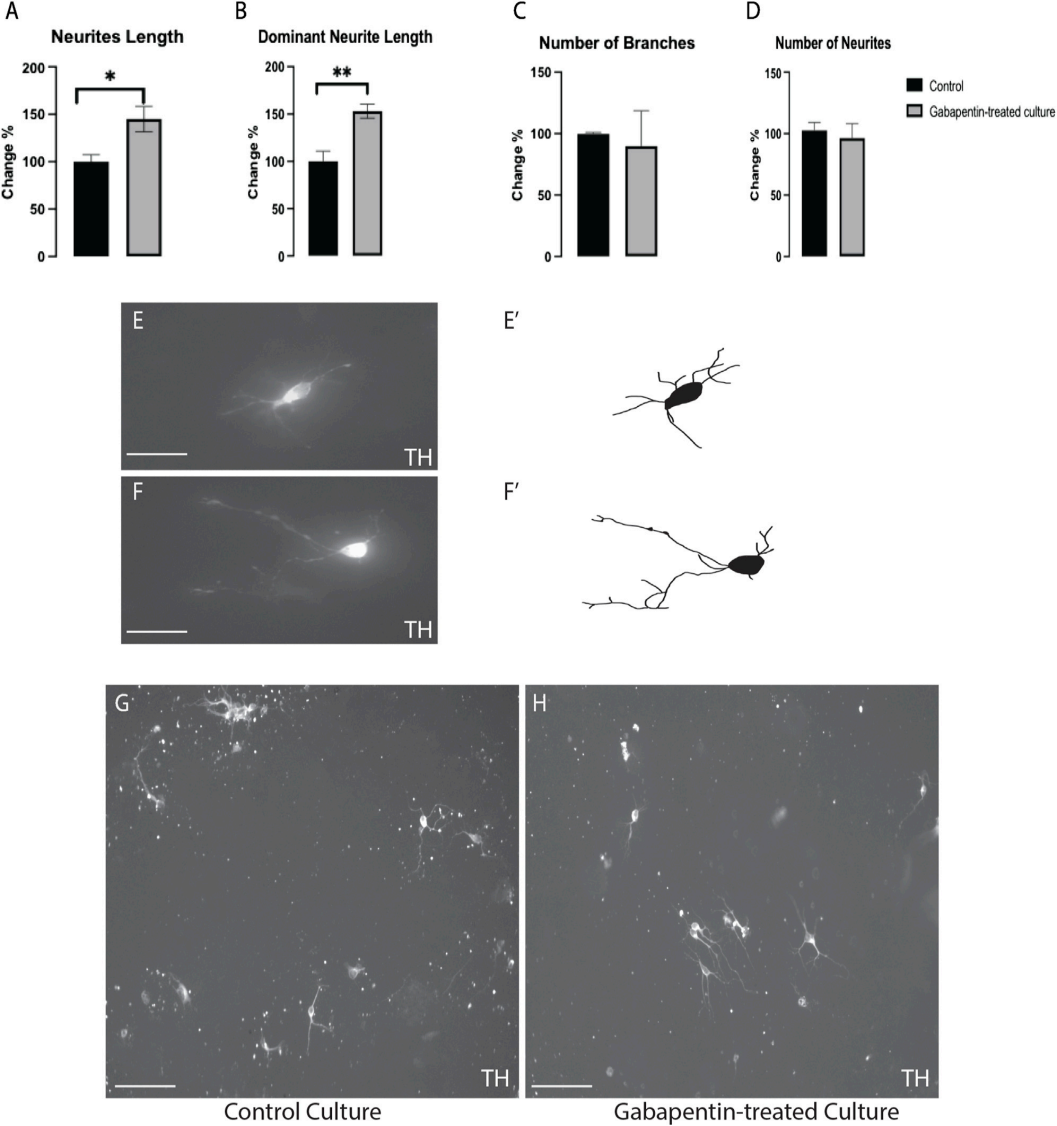
Gabapentin-induced elongation observed in total neurite length **(A)** and dominant neurite length **(B)** of TH + vmDA neurons. TH + vmDA neurons showing no significant changes in the number of branches **(C)** and neurites **(D)** of in response to gabapentin treatment. Representative images and illustration for vmDA neurons immunolabeled with TH in both groups; control **(E,E’)** and gabapentin-treated **(F,F’)** show the increase in neurites elongation in response to gabapentin exposure. **(G,H)** images show large field of view (20x) for both groups; control and gabapentin treated cultures, respectively. Scale bar = 50 um **(E,F)**. Scale bar = 10 um **(G,H)**. Data are represented as mean ± SEM, *n* = 4 experiments. *p < 0.05.

The impact of gabapentin on vmDA TH + neuronal morphogenesis was investigated in immune-stained cultures. We found a significant increase in the TH + total neurite length (Figure 3A) and dominant neurite length (Figure 3B) in gabapentin-treated cultures in comparison with the lengths observed in the control. However, the number of branches (Figure 3C) or neurites (Figure 3D) of TH + vmDA neurons in the gabapentin-treated cultures did not differ significantly from those in the control cultures (Figures 3E–H).

### Gabapentin Affects the Morphogenesis of VM Non-DA Neurons

**FIGURE 4:**
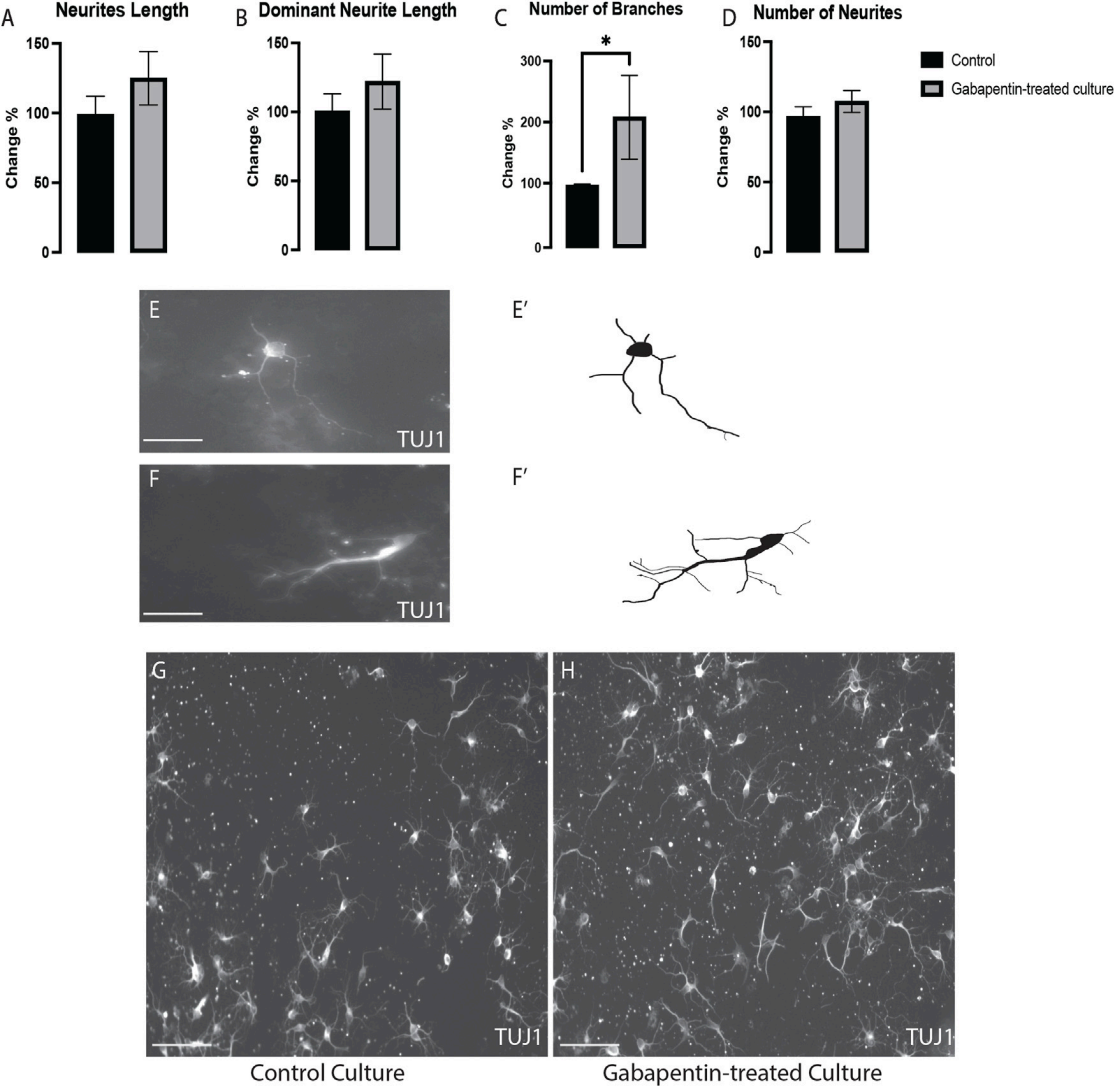
VM nondopaminergic neurons (TUJ1+/TH-) subjected to gabapentin exposure having no changes in the total neurite length **(A)**, the dominant neurite length **(B)** or the number of neurites **(D)**, while having significant increases in the number of branches **(C)**. Representative images and illustration for VM neurons immunolabeled with TUJ1 in both groups; control **(E,E’)** and gabapentin-treated **(F,F’)** show the increase in the numbers of branches in response to gabapentin exposure. **(G,H)** images show large field of view (20x) for both groups; control and gabapentin treated cultures, respectively. Scale bar = 50 um **(E,F)**. Scale bar = 10 um **(G,H)**. Data are represented as mean ± SEM, *n* = 4 experiments. *p < 0.05.

To determine if gabapentin affects the morphogenesis of all VM neurons or specifically that of vmDA neurons, we investigated the effect of gabapentin on VM non-DA (TUJ1+/TH-) neurons. We detected a significant increase in the number of branches of TUJ1+/TH- neurons (Figure 4C). However, no significant differences were observed in the total neurite length (Figure 4A), dominant neurite length (Figure 4B), or the number of neurites (Figure 4D) in TUJ1+/TH- neurons between the treatment and control cultures (Figures 4E–H). These observations reveal that the alteration of the morphogenesis of nondopaminergic and dopaminergic VM neurons was affected differently by gabapentin.

### Gabapentin Induces the Upregulation of Key Dopaminergic-Related Genes in vmDA Neurons

**FIGURE 5:**
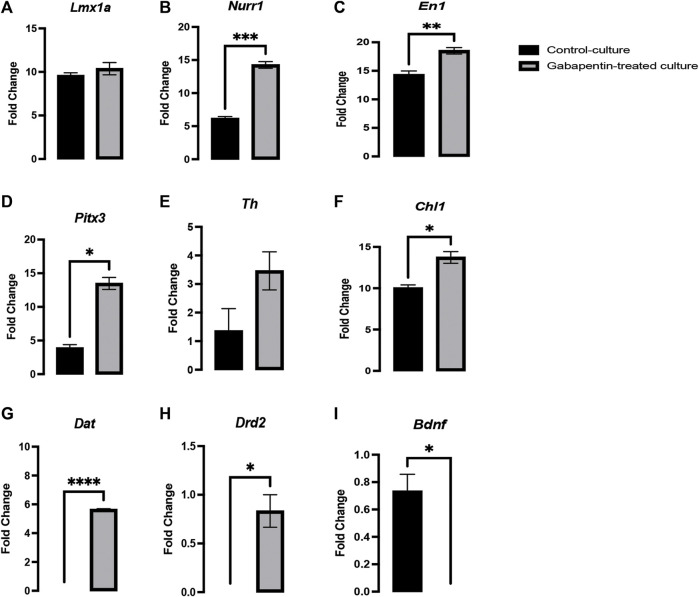
Gabapentin exposure inducing a significant upregulation in the expression of *Nurr1* **(B)**, *En1* **(C)**, *Pitx3* **(D)**, *Chl1* **(F)**, *Dat* **(G)** and *Drd2* **(H)**, significant downregulation the expression of *Bdnf* **(I)**, and no significant change in the expression of *Lmx1a* **(A)** and *Th* **(E)** in vmDA neurons.

Several genes involved in neurogenesis have been discovered, such as *Lmx1a/b*, *Mash1*, and *Ngn2*, which play a role in early DA fate, *Wnt5a/7a* and *Netrin1*, which are involved in axonal plasticity, and *Wnt5a*, *Pitx3*, and *Th*, which play an important role in neuronal maturation. However, all these genes fail to encompass all activities associated with vmDA neuronal development (Burbach et al., 2003; Van den Heuvel and Pasterkamp, 2008; Fernando et al., 2014; Parish and Thompson, 2014). The fate and functional activities of mDA progenitors involve genes such as *Lmx1a/b*, which play an important role in neurogenesis (Doucet-Beaupré et al., 2016). Both *Lmx1a* and *Lmx1b* are closely associated and have 64% similarity in their amino acid sequence, 67 and 83% identity in the LIM domain, and 100% similarity in their homeodomain (Hobert and Westphal, 2000). The gabapentin-treated cultures in our experiments showed no significant change in *Lmx1a* expression compared to that in control cultures (Figure 5A). Genes such as *Lmx1a*, *Nurr1*, and *Mash1* are associated with a minimal transcription factor mixture and enable vmDA neurons to be generated directly from murine and human fibroblasts without reversion to a progenitor cell stage (Caiazzo et al., 2011). Our results showed a significant upregulation in the expression of *Nurr1* and *En1* in the gabapentin-treated cultures (Figures 5B,C). Several studies have reported genes such as *Nurr1*, *En1*, *Pitx3*, *Otx2*, and *Foxa2* to be involved in early development of vmDA neurons and are crucial for the maintenance phenotype of neurons (Doucet-Beaupr and Lvesque, 2013). Our results demonstrated a significant upregulation in the expression of *Pitx3* in response to gabapentin exposure *in vitro* (Figure 5D). Studies have claimed that *Nurr1* is activated by *Lmx1a* and subsequently activates *Th*, which is associated with vmDA neuronal differentiation (Bergman et al., 2009; Hoekstra et al., 2013). Although the expression of the maturation-related gene *Pitx3* was altered in response to gabapentin exposure, the expression of *Th* was not significantly changed in the treated cultures in comparison with that in the control (Figure 5E). The expression profile of *Pitx3* suggested that gabapentin could alter vmDA neuronal maturation. Previous studies by our group, and by others, have reported the involvement of *Chl1* in vmDA development (Hoekstra et al., 2013; Alsanie et al., 2017). Our current results showed a significant upregulation in the expression of *Chl1* (Figure 5F), which suggests that gabapentin may interfere with the neurogenesis and maturation of vmDA neurons. Our observations suggested that gabapentin follows the *Chl1/En1/Nurr1/Pitx3* pathway for neuronal differentiation. The downstream targets of *Nurr1*, such as *Bdnf*, *Dat*, and *Drd2*, are highly important for vmDA maturation and neurogenesis (Johnson et al., 2011; Hegarty et al., 2013; Kadkhodaei et al., 2013; Torretta et al., 2020). The expression of these downstream targets was significantly altered in response to gabapentin treatment. The expression of *Dat* and *Drd2* was significantly upregulated in gabapentin-treated cultures in comparison with that in the control (Figures 5G,H). However, *Bdnf* was significantly downregulated in response to gabapentin exposure (Figure 5I). These changes in the expression of the key genes might negatively affect the normal development of vmDA neurons.

## Discussion

Gabapentin is a GABA analog, which is commonly prescribed for treatment of neuropathic pain. However, it does not have an affinity for GABA receptors. Gabapentin increases GABA levels and decreases those of glutamate neurotransmitters. The neural population plays a major role in behavior, cognition, and motor activity. The fate of vmDA neurons is determined by intrinsic as well as extrinsic signaling ques. To establish a mature state and a final resting place, the neurons respond to several signals pertaining to neuronal connectivity during the cell cycle. Similar to the other neural populations, VM neurons are known to have early and late developmental stages. Our recent investigation hypothesized that gabapentinoids could affect the dopaminergic neuronal system (Althobaiti et al., 2020, 2021). In the current study, we observed that gabapentin at therapeutic doses has no impact on the survivability of VM neurons. However, our results also showed that there was a significant reduction in ATP release in VM neurons. Therefore, this study reveals that gabapentin could negatively impact the early stage of vmDA neurogenesis and morphogenesis. These results are important for the design of preclinical studies to determine the safety of gabapentin use during pregnancy.

Transcription factors such as *Nurr1*, *Pitx3*, and *Lmx1a* are critical for midbrain specification. We found that gabapentin could induce high expression of dopaminergic related genes, which suggested that gabapentin use might not be safe during pregnancy. Gabapentin is commonly used as a first-line treatment and is the most frequently prescribed drug in neuropathic disorders. The misuse of gabapentin is known to cause serious negative effects. Thus, several studies have been focused on investigating its safety in child-bearing women. The mechanism by which gabapentin interferes in fetal brain development is still not clear (Patorno et al., 2020). Antiepileptic drugs such as valproate have demonstrated adverse neurological effects when used alone or combined with other antiepileptics. Lamotrigine and oxcarbazepine have been associated with an increased occurrence of autism (Veroniki et al., 2017). This has made it necessary to develop optimum treatments with safe regimens (Veroniki et al., 2017). Previous studies have reported that large randomized clinical trials are required to confirm whether gabapentin does not interfere with neuronal brain development and is therefore safe to be prescribed for pregnant women (Guttuso et al., 2014). Hence, in the present study, we examined the impact of gabapentin on the development of vmDA neurons, which play an important role in rewards, motor activity and addiction. (Hegarty et al., 2013).

We treated primary mouse embryonic vmDA neurons with gabapentin to examine their survivability. We observed that gabapentin exposure had no impact on viability of vmDA neurons, confirming that gabapentin is nontoxic at therapeutic doses. Previous studies on the use of antiepileptics during pregnancy have concluded that valproic acid, lamotrigine, and carbamazepine are associated with smaller head circumference observed in the fetus (Margulis et al., 2019). Moreover, another study showed that gabapentin administration during pregnancy is associated with adverse gestational outcomes (Loudin et al., 2020). Therefore, the safety of the usage of gabapentin during pregnancy has not yet been conclusively determined (Loudin et al., 2020). Thus, antiepileptics, such as gabapentin are generally prescribed according to the risk-to-benefit ratio (Guttuso et al., 2014; Patorno et al., 2020). In the current study, we showed that gabapentin altered vmDA neuronal metabolic activity by decreasing the generation of ATP. These findings confirmed that gabapentin disrupts the activities of the electron transport chain, leading to ATP deficiency. Furthermore, we assessed the effect of gabapentin on the morphogenesis of vmDA neurons by investigating changes in the total and dominant neurite lengths following gabapentin treatment. We are reporting here that gabapentin treatment increased TH + vmDA neurites length (total and dominant neurites length). On the other hand, the numbers of neurites and branches were not significantly different between those in the treated and control groups. Contrarily, in the case of VM non-DA neurons, gabapentin significantly increased the number of branches, while the length of neurites remained the same. These findings are similar to those shown previously in our study on pregabalin, which is another drug belonging to the gabapentinoid family (Alsanie et al., 2022). However, pregabalin increased the number of neurites of VM non-DA neurons, an effect that was not detected with gabapentin treatment. Importantly, the alteration in the morphogenesis of vmDA neurons could disrupt the connectivity of these neurons with their neuronal targets. Consequently, these changes could lead to neurological disorders by abnormally changing behavior, cognition and/or motor activity.

Many genes such as *Lmx1b, En1, Nurr1, Pitx3,* and *Lmx1a*, are known to play a vital role in the development of vmDA neurons. (Yin et al., 2009; Volpicelli et al., 2012; Hoekstra et al., 2013). Previous studies have illustrated that *Lmx1a and Nurr1* activate *Th,* which ultimately helps in vmDA neuronal differentiation and maturation (Bergman et al., 2009; Hoekstra et al., 2013). *Pitx3* is regulated by *Rspo2,* which is activated by *Lmx1a* (Gyllborg et al., 2018)*.* Considering these findings, we investigated the effects of gabapentin on the key genes associated with the development of vmDA neurons. We found that gabapentin significantly increased *Nurr1, Chl1, En1, Bdnf, Drd2* and *Pitx3* expression (Figure 5). Contrarily, no changes were detected in the expression profile of *Lmx1a* and *Th*. These observations suggested that gabapentin follows the *Nurr1, En1, Chl1* and *Pitx3* signaling pathways. Former studies have also showed that the expression of several major genes, such as *Bdnf*, *Drd2,* and *Dat,* is regulated by *Nurr1* (Johnson et al., 2011; Hegarty et al., 2013; Kadkhodaei et al., 2013; Torretta et al., 2020). *Bdnf* is known to be a trophic factor for vmDA neurons and is therefore critical for their viability (Hyman et al., 1991).*Drd2* and *Dat* are crucial genes for the development, maturation and functionality of vmDA neurons (Sung et al., 2006; Hwang et al., 2009; Hegarty et al., 2013). In the present study, we demonstrated that *Drd2* and *Dat* are upregulated significantly by gabapentin, which may be attributed to high *Nurr1* expression, which is also similar to that showed in our earlier study on pregabalin (Alsanie et al., 2022). A former study has illustrated that gabapentin exposure increases the expression of *Pitx3,* which in turn stimulates *Dat* in coordination with *Nurr1* (Hwang et al., 2009). The high expression of *Drd2* due to gabapentin exposure might be the reason behind the reported increase in the neurite length of TH + neurons. The elongation of dopaminergic neurons induced by DRD2 receptors has also been reported in an earlier study by Hwang and colleagues (Hwang et al., 2009). Although *Bdnf* is one of the downstream targets of *Nurr1*, our results showed that the expression of *Bdnf* was significantly downregulated by gabapentin treatment. Previously, we demonstrated that the expression of *Bdnf* was upregulated by pregabalin treatment in vmDA neuron cultures (Alsanie et al., 2022). Although gabapentin and pregabalin are both gabapentinoids, their effects on vmDA-related genes are thus shown to be different. Such alternate expression of the vital genes has been reported to be associated with several psychological, mental and neurological problems (Hansen et al., 2014; Zhang and Zhang, 2016; Tsai, 2018).

## Conclusion

Our study examined the effect of gabapentin treatment on vmDA neurons, which are in charge of cognition, motion, and behavior. We used primary mouse embryonic VM neurons to investigate the impact of prenatal exposure to gabapentin on embryonic vmDA development. Our results revealed that gabapentin exposure during neurodevelopment interfered with the neurogenesis and morphogenesis of vmDA neurons. Our findings suggest that gabapentin usage in pregnant women, even at therapeutic levels, interferes with the neurogenesis and morphogenesis of vmDA neurons during fetal brain development. Furthermore, several genes involved in the influence of gabapentin on vmDA development were identified. These changes could eventually alter the functionality of vmDA neurons. These findings have important implications on the safety assessment of the therapeutic use of gabapentin during pregnancy. Future *in vivo* and neuronal functionality studies can be conducted to better evaluate the effects of gabapentin on vmDA neurons. In addition, neuronal cultures established from human embryonic stem cells could be used to further investigate the effects of gabapentin exposure on human neurons.

## Data Availability

The original contributions presented in the study are included in the article/Supplementary materials, further inquiries can be directed to the corresponding author.
